# Metastasis from follicular lymphoma to an ovarian mature teratoma: a case report of tumor-to-tumor metastasis

**DOI:** 10.1186/s13048-023-01188-0

**Published:** 2023-05-31

**Authors:** Yusuke Sato, Mitsutake Yano, Satoshi Eto, Kuniko Takano, Kaei Nasu

**Affiliations:** 1 Department of Obstetrics and Gynecology, Faculty of Medicine, Oita University, Oita, Japan; 2 Department of Medical Oncology and Hematology, Faculty of Medicine, Oita University, Oita, Japan; 3 Division of Obstetrics and Gynecology, Support System for Community Medicine, Faculty of Medicine, Oita University, Oita, Japan

**Keywords:** Tumor-to-tumor metastasis, Lymphoma, Ovarian mature teratoma, Case report, Molecular analysis, Malignant transformation

## Abstract

**Background:**

Tumor-to-tumor metastasis (TTM) is a rare but well-established phenomenon where histologically distinct tumors metastasize within each other. Here we report the first “known” case of follicular lymphoma that metastasized and extended to a mature ovarian teratoma.

**Case presentation:**

A 59-year-old Japanese postmenopausal woman visited our hospital for a detailed examination of an ovarian tumor. Clinical imaging suggested it to be either teratoma-associated ovarian cancer with multiple lymph node metastases, or tumor-to-tumor metastasis from malignant lymphoma to ovarian teratoma. A bilateral adnexectomy and retroperitoneal lymph node biopsy were performed. Lined with squamous epithelium, the cyst constituted a mature ovarian teratoma, and the solid part showed diffuse proliferation of abnormal lymphoid cells. Immunohistochemically, the abnormal lymphoid cells were negative for CD5, MUM1, and CyclinD1, and positive for CD10, CD20, CD21, BCL2, and BCL6. Genetic analysis using G-banding and fluorescence in situ hybridization identified a translocation of t(14;18) (q32;q21), and we diagnosed tumor-to-tumor metastasis from nodal follicular lymphoma to mature ovarian teratoma. Twelve months after surgery, the patient showed no progression without adjuvant therapy.

**Conclusions:**

The present case suggests that molecular approaches are useful in the diagnosis of TTM in mature ovarian teratomas when morphologic and immunohistochemical findings alone are insufficient for diagnoses.

## Background

Ovarian mature teratomas (OMTs), which are composed of cells derived from two or three germ cell layers, are among the most common benign ovarian tumors. Malignant transformation of OMTs causes various cancers, such as squamous cell carcinoma (SCC), adenocarcinoma, neural tumor, malignant melanoma, and lymphoma [1–3]. The ovaries can also receive cancer metastasis from other organs [4]. Therefore, when cancers are found in OMTs, it is difficult to determine whether they are due to malignant transformation (primary) or metastasis.

Tumor-to-tumor metastasis (TTM) is a rare but well-established phenomenon defined as metastasis in histologically distinct tumors [5]. To date, three cases of TTM to OMTs have been reported: appendiceal adenocarcinoma [5], cervical adenocarcinoma [6], and breast cancer [3]. Ten cases of primary lymphoma derived from OMTs [7, 8] and only one cases of lymphoma-led invasions into OMTs are reported [9]. Here, we report a case of coexisting ovarian maturing teratoma and follicular lymphoma, diagnosed as TTM, using morphological, immunohistochemical, and molecular approaches.

## Case presentation

**Fig. 1:**
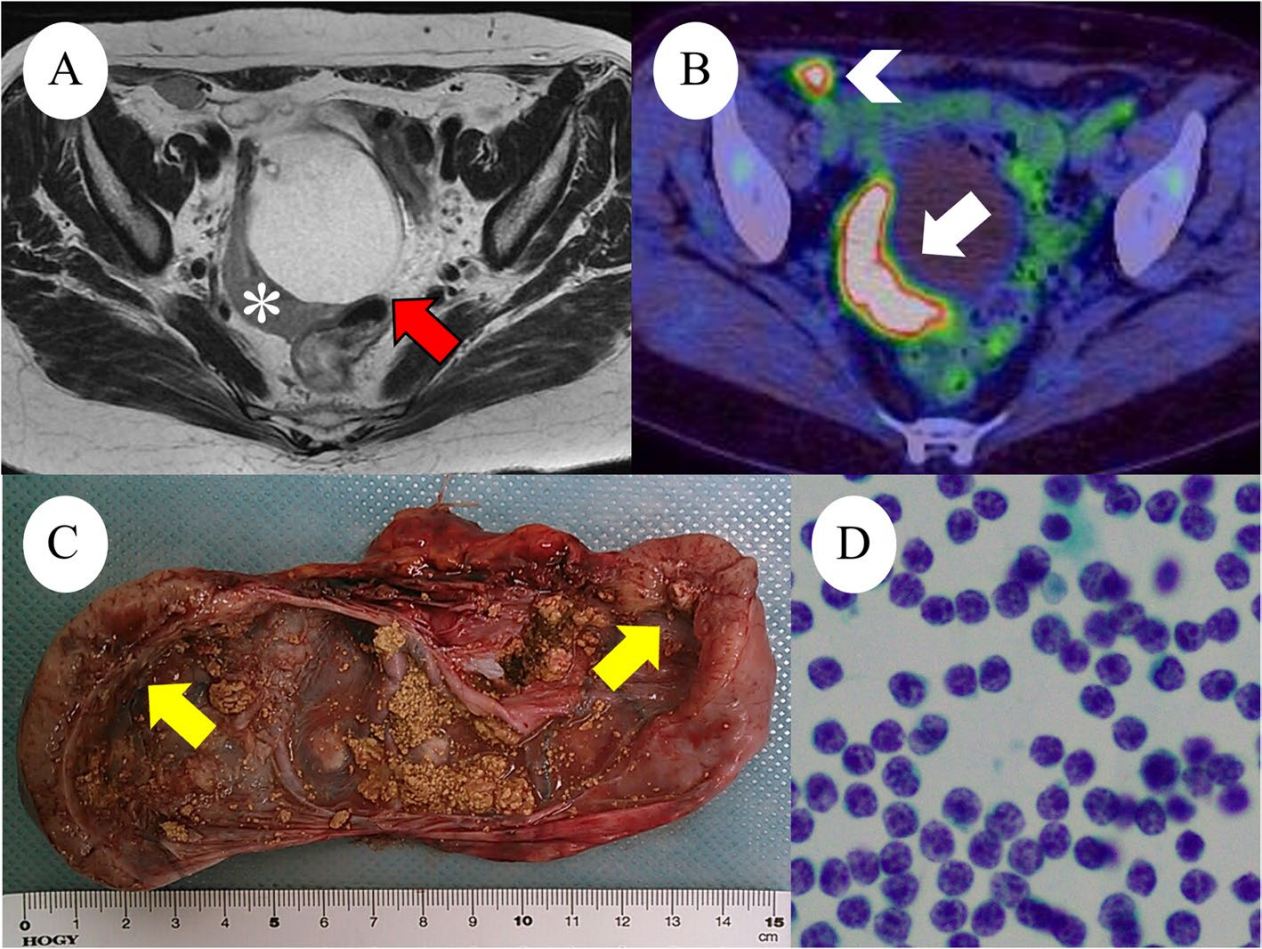
**A**: Axial T2-weighted magnetic resonance image reveals a 70-mm right ovarian mass (red arrows) with a 20-mm crescent solid part at the dorsal side (white asterisks). **B**: Positron emission tomography-CT shows FDG accumulation in the solid component (white arrows), measuring SUV max od 8.3 and retroperitoneal lymph node (white arrowhead), measuring SUV max of 7.8. **C**: Right ovarian tumor, macroscopic examination shows cystic tumor filled with fatty and yellow sebaceous component and solid nodule (yellow arrows). **D**: Ascites cytology was no neoplastic findings

**Fig. 2:**
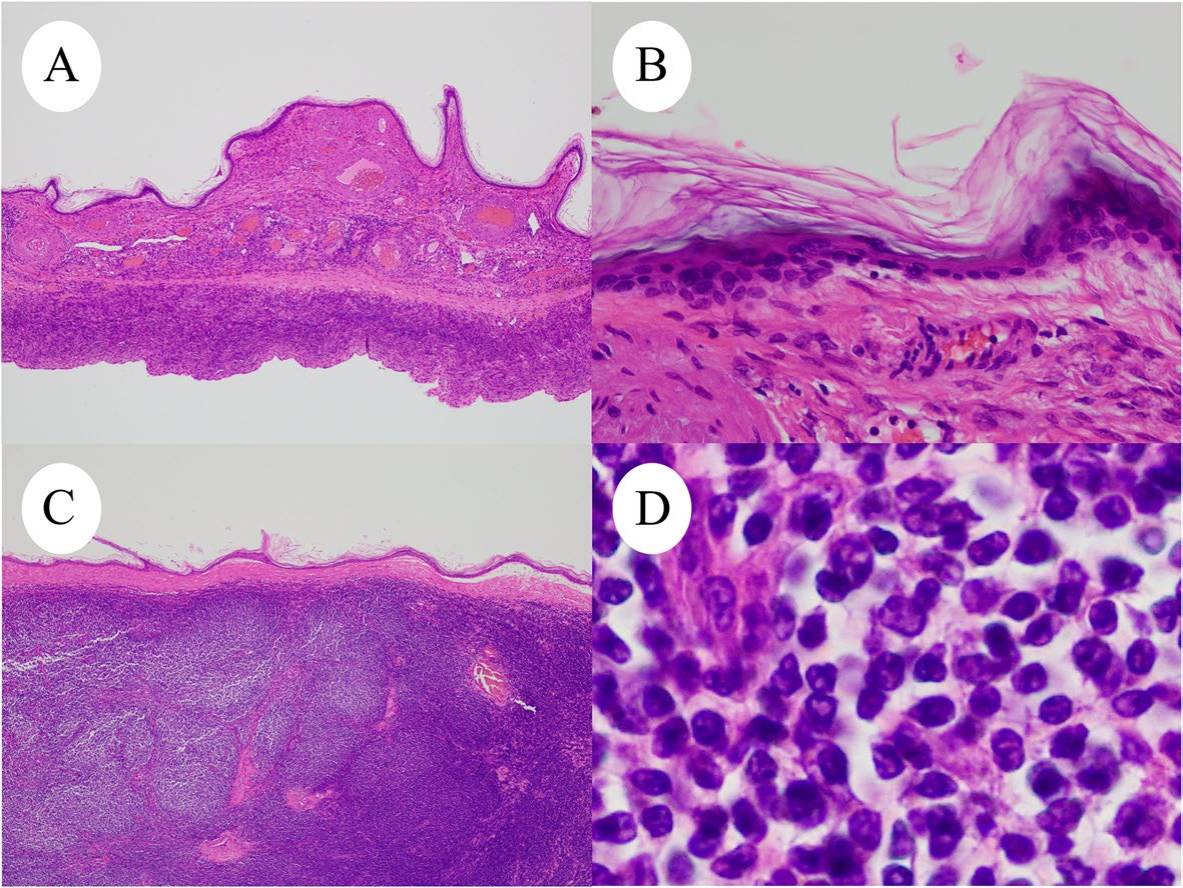
Microscopic examination of right ovarian tumor (H&E). **A** and **B**: Cystic wall covered with keratinized squamous epithelium. **C** and **D**: Microscopic examination shows the solid nodule consisted of diffusely proliferated abnormal lymphoid cells

**Fig. 3:**
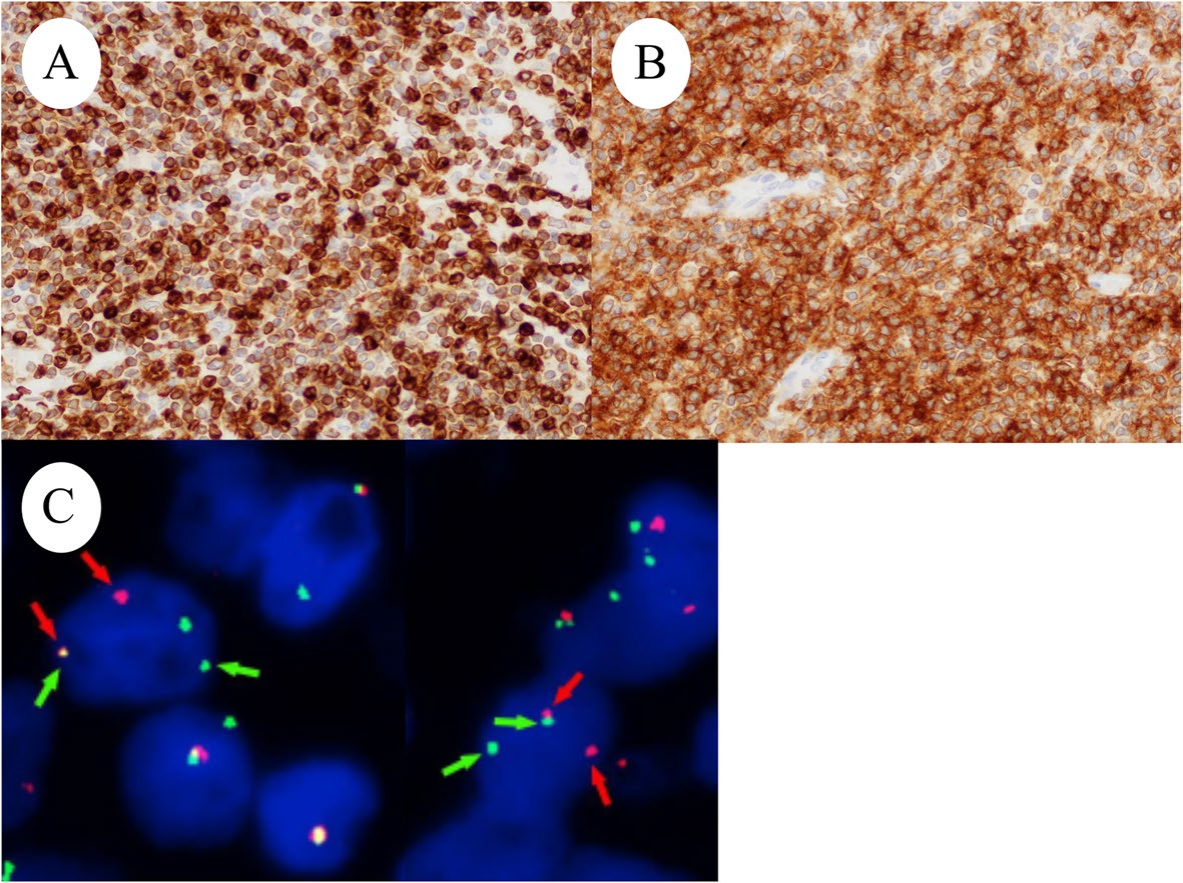
Right ovarian tumor is positive for (**A**) BCL2 and (**B**) CD10. (**C**) FISH analysis. Red probes the centromere side of the BCL gene (18q21) breaking point, and green probes the telomeric side. Split signals were observed

A 59-year-old Japanese postmenopausal woman (gravida 2, para 2) with no history of being immunocompromised, visited our hospital for a detailed examination of an ovarian tumor found during a medical checkup. She had a family history of prostate and pharyngeal cancer in her father and mother, respectively. Transvaginal ultrasonography showed a 70-mm cystic tumor with solid parts in the right ovary. Contrast-enhanced computed tomography and plain magnetic resonance imaging (MRI) showed a right ovarian teratoma and multiple enlarged retroperitoneal lymph nodes (Fig. 1A). 18 F-fluorodeoxyglucose-positron emission, tomography-computed tomography revealed increased fluorodeoxyglucose accumulation in the right ovarian tumor and pelvic to axillary lymph nodes (Fig. 1B). These clinical images suggested OMT-associated ovarian cancer with multiple lymph node metastases or TTM from malignant lymphoma to OMT. Carbohydrate antigen 125 level of 32.9 U/mL (normal range, ≤ 35.0 U/mL), carbohydrate antigen 19 − 9 level of 3.0 U/mL (normal range, ≤ 37.0 U/mL), and soluble Interleukin-2 receptors level of 432 U/mL (normal range, 204–587 U/mL) were normal. Soluble interleukin-2 receptors are one of the useful serum markers for lymphoma. A bilateral adnexectomy and retroperitoneal lymph node biopsy were performed, revealing a moderate amount of milky ascites. Macroscopically, the right ovarian tumor was a cystic mass with fat and solid parts (Fig. 1C). Ascitic cytology revealed no neoplastic (Fig. 1D). The cyst was lined by squamous epithelium constituting an OMT, and the solid part showed diffuse proliferation of abnormal lymphoid cells (Fig. 2A-D). Immunohistochemically, abnormal lymphoid cells of the ovarian tumor tested negative for CD5, MUM1, and CyclinD1, and positive for CD10, CD20, CD21, BCL2, and BCL6 (Fig. 3A-B). The abnormal lymphoid cells of the retroperitoneal lymph nodes were negative for CD5, CMYC, MUM1, and CyclinD1, and positive for CD10, CD20, CD21, CD79a, BCL2, and BCL6. Genetic analysis of the lymphoma using G-banding and fluorescence in situ hybridization (FISH) identified a translocation of t(14;18) (q32;q21) (Fig. 3C) and diagnosed TTM from nodal follicular lymphoma (histological grade 1) to OMT. Twelve months after the surgery, the patient showed no recurrence without adjuvant therapy.

## Discussion

TTM is defined as metastasis in distinct tumors of the same body, and 15 cases of TTM in ovarian tumors have been reported to date [5]. OMTs causes various cancers via malignant transformation; therefore, the coexistence of OMT and cancers requires the differentiation between primary and metastatic cancers.

Ten cases of primary malignant lymphoma derived from OMT have been reported [7, 8]. TTM of malignant lymphoma to OMT is the only existing case of plasmablastic lymphoma [9]. TTM to OMT is summarized in Table 1 [3, 5, 6, 9] and shows that the present case is the first recorded case of nodal follicular lymphoma metastasizing and extending to the OMT. Historically, the most common method for diagnosing TTM to OMT was immunohistochemical analysis (4/5 cases), followed by virological examination and FISH, which were performed in two cases each. This is the first molecular analysis report that combines G-banding and FISH. These results recommend molecular diagnostic approaches for TTM and OMT when morphological and immunohistochemical findings alone are insufficient for diagnoses.

**Table 1:** Tumor-to-tumor metastasis to mature ovarian teratoma

Case (years)	Age	Metastasis From	Differential diagnosis method				References
	(years)		IHC	Virology	G-banding	FISH	
The present case	59	Follicular lymphoma	Yes	No	Yes	t(14;18)(q32;q21)	Not applicable
Yano M (2019) [5]	67	Appendiceal adenocarcinoma	Yes	No	No	No	#5
Santos F (2018) [6]	51	Cervical adenocarcinoma	Yes	HPV	No	No	#6
Hadžisejdić I (2017) [9]	19	Plasmablastic lymphoma	Yes	EBV	No	8q24 rearrangement	#9
Kirova YM (1999) [3]	47	Breast carcinoma	No	No	No	No	#3

Abbreviations: *IHC*, Immunohistochemistry, *FISH *Fluorescence in situ hybridization, *EBV *Epstein-Barr virus, *HPV *Human papillomavirus

Ozsan et al. divided 16 follicular lymphomas initially diagnosed in the ovary into two groups based on clinical, morphological, immunophenotypic, and genetic features [10]. Ozsan et al. reported that ovarian extension of nodal follicular lymphoma was characterized as low histologic grade, BCL2 protein positivity, and presence of t(14;18)(q32;q21), while true primary ovarian follicular lymphoma was characterized as higher histologic grade, CD10 and BCL2 negativity, and absence of t(14;18)(q32;q21) [10]. We diagnosed the present case with TTM from nodal follicular lymphoma to OMT based on the distribution of lymph node lesion (pelvic to axillary), genetic analysis of t(14;18)(q32;q21), immunohistochemical expression of BCL2 and CD10, and histologic grades (grade 1). However, this study included only one case of OMT [10]. It is unclear whether OMT-derived follicular lymphoma has the same biology as that of a conventional follicular lymphoma. Tamura et al. reported that SCCs derived from OMTs share genetic characteristics with pulmonary SCCs rather than cutaneous SCCs, which is the most common component of OMTs [11]. It is expected that more cases will be accumulated in the future.

## Conclusion

To the best of our knowledge, the present case is the first report of follicular lymphoma metastasizing and extending to the OMT. To make this differential diagnosis of TTM and primary ovarian follicular lymphoma, we used immunohistochemical and molecular analyses (G-banding and FISH) for the first time.

## Data Availability

The datasets used and/or analyzed during the current study are available from the corresponding author upon reasonable request.
